# Blood Pressure Variability Is Associated with Hearing and Hearing Loss: A Population-Based Study in Males

**DOI:** 10.1155/2019/9891025

**Published:** 2019-02-03

**Authors:** Minghui Bao, Yongjian Song, Jun Cai, Shouling Wu, Xinchun Yang

**Affiliations:** ^1^Department of Heart Center, Chaoyang Hospital, Capital Medical University, Beijing, China; ^2^Graduate School, North China University of Science and Technology, Tangshan, China; ^3^Department of Cardiology, Kailuan Hospital, North China University of Science and Technology, Tangshan, China; ^4^Hypertension Center of Fuwai Hospital, State Key Laboratory of Cardiovascular Disease, National Center for Cardiovascular Diseases, Chinese Academy of Medical Sciences and Peking Union Medical College, Beijing, China

## Abstract

Blood pressure (BP) has been well documented to be associated with hearing loss previously. However, the role of blood pressure variability (BPV, representing BP fluctuation over a time period) on hearing remains unknown. We aimed to evaluate the relationship between BPV and hearing in Chinese population. We included 8646 male subjects from a population-based study (the Kailuan study). BP was measured every two years at routine physical examinations from 2006 to 2015. Based on five annual BP measurements, BPV was estimated by standard deviation of BP (SD), coefficient of the variation of BP (CV), and variation independent of mean of BP (VIM). Hearing was estimated by pure-tone average threshold (PTA) at low, intermediate, and high frequencies in the year of 2014. Regression models were used to evaluate the relationship between BPV and hearing. The results showed that PTAs and percentages of hearing loss at low, intermediate, and high frequencies grew gradually with increasing systolic SD (SSD) (p<0.05). After adjusting for multiple covariates, multivariate regression analyses demonstrated that variations of SBP (SSD, SCV, and VIM_SBP_) were all positively correlated with PTA at intermediate and high frequencies (p<0.05). Each SD increase in SSD, SCV, and VIM_SBP_ was also positively associated with hearing loss at intermediate and high frequencies. No significant correlation was observed between variations of DBP and hearing. These findings suggest that increase in long-term BPV is associated with hearing and hearing loss.* Trial registration number:* Kailuan study (ChiCTRTNC-11001489).

## 1. Introduction

Hearing loss ranks as the fifth leading cause of years lived with disability, affecting 360 million people worldwide [1]. While not life-threatening, hearing loss negatively influences the quality of life, physical function, and psychosocial well-being of individuals [2–6]. Multiple congenital and acquired causes can lead to hearing loss, such as ageing, noise exposure, using ototoxic drugs, genetic alterations, and systemic diseases.

Hypertension is a major global health burden and a leading risk factor for cardiovascular diseases and premature death [7–11]. However, growing evidence suggests that blood pressure (BP) values alone may not fully explain the pathophysiological relationship between BP and adverse cardiovascular events. Post hoc analyses of clinical trials and observational studies indicate that blood pressure variability (BPV), defined as the extent of BP fluctuation over a time period, is associated with cardiovascular diseases [12–14]. Several studies have reported relationships between BPV and cardiovascular events [15, 16], mortality, and end-organ damage [17–19]. Moreover, recent data suggest that visit-to-visit variability over relatively long follow-up periods (e.g., month-to-month and year-to-year) has greater prognostic value than average BP or BPV over short follow-up periods (e.g., minute-to-minute and hour-to-hour) [20, 21], leading to increased interest in the prognostic importance of long-term BPV.

Clinical and experimental studies have demonstrated that arterial hypertension was an independent risk factor for the hearing loss. Patients with higher BP had worse pure-tone thresholds [22–25]. Exposure to high-frequency noise can cause much greater loss of cochlear hair cells in spontaneously hypertensive (SH) rats than in normotensive ones [26, 27]. Although the impact of hypertension on hearing has been extensively studied, the relationship between BPV and hearing level or hearing loss has never been reported by previous researches. Therefore, on the basis of the population of the Chinese Kailuan study (ChiCTR-TNC-11001489), we adopted pure-tone average thresholds (PTAs) and hearing loss as indexes to investigate whether BPV can affect cochlear function of individuals.

## 2. Materials and Methods

### 2.1. Study Participants

This study was performed based on the Kailuan Community in Tangshan. Physical examinations were conducted every two years on both in-service and retired workers of Kailuan Community. Eleven hospitals participated in the physical examination. A total of five physical examinations were performed during 2006-2007, 2008-2009, 2010-2011, 2012-2013, and 2014-2015, respectively. The measurement of PTAs was performed in 2014-2016.

### 2.2. Inclusion and Exclusion Criteria

The inclusion criteria were as follows: giving signed informed consent to participate in the current study, providing complete information from at least three of the five physical examinations, and providing complete information of PTA measurements. The exclusion criteria were as follows: history of stroke, history of head injury, history of myocardial infarction, history of atrial fibrillation, missing BP data from more than two of the five physical examinations, missing data of the measurements of PTA, and female individuals (female individuals were ruled out because the sample size of female was too small compared to male subjects and the unbalanced gender distribution may result in gender bias).

### 2.3. Data Collection

#### 2.3.1. Epidemiological Questionnaire

The questionnaire was completed by individuals and then verified by research doctors. The questionnaire items were consisted of demographic information, occupation situation (the questionnaire items about occupation situation were consisted of type of occupation and nature of work (mental work or physical work), employing conditions (serving or retired), labor intensity (extremely light, light, intermediate, and heavy), occupational hazard (e.g., noise, high temperature, and microwave, harmful chemicals, and dust exposure)), lifestyle (e.g., cigarette smoking, exercise, and diet), disease history and family history, and physical examination profiles (e.g., blood pressure, height, weight, waist circumference, etc.). Smoking was defined as ≥1 cigarette/day, continuous smoking ≥1 year, or giving up smoking ≤1 year. Alcohol consumption is defined as continuous drinking≥1 year (alcohol content >50%, amount >100ml). Physical exercise is defined as aerobic exercise (such as walking, jogging, ball games, and swimming) ≥3 times/weeks and ≥ 30min/times. Occupational noise exposure is defined as working places equivalent environmental noise level ≥85 decibel (dB) at least 8 hours per day.

#### 2.3.2. Anthropometric and Biochemical Measurements

Standard protocols were used for all of the measurements as described earlier by our group [28]. Anthropometric measurements included the measurements of height, weight, body weight index (BMI), and blood pressure (BP). Biochemical measurements included fasting plasma glucose (FBG), triglycerides (TG), total cholesterol (TC), high-density lipoprotein cholesterol (HDL-C), and low-density lipoprotein cholesterol (LDL-C). Blood samples were collected from the antecubital vein in the morning after an overnight fast and transferred into EDTA-containing vacuum tubes. FBG was measured by the hexokinase method. TC and triglycerides were measured enzymatically (interassay coefficient of variation <10%; Mind Bioengineering Co., Ltd., Shanghai, China). Diabetes mellitus was defined as FBG ≥7.0 mmol/L and/or FBG <7.0 mmol/L with regular antidiabetics usage [29]. Lipid abnormalities were defined as TC > 5.0 mmol/L or LDL-C > 3.0 mmol/L or TG > 1.7 mmol/L [30]. Individuals were categorized into two groups according to the BMI levels. BMI < 24 kg/m^2^ was defined as normal weight, while BMI ≥ 24 kg/m^2^ was defined as overweight [31]. All biochemical variables were measured using an automatic analyzer (Hitachi 7600 automatic analyzer) at the central laboratory of the Kailuan General Hospital.

#### 2.3.3. BP Measurement

BP was measured between 7:00 and 9:00. Individuals were asked to refrain from smoking and drinking tea or coffee for more than 30 min and to sit and rest for 15 min prior to measurement. During BP measurement, individuals sat with their arms and feet flat and their upper arms at the height of their heart. Right brachial artery BP was measured by a corrected mercury sphygmomanometer with an appropriate sized cuff. Systolic blood pressure (SBP) was recorded on hearing the phase I Korotkoff sound. Diastolic blood pressure (DBP) was recorded on hearing the phase V Korotkoff sound. Sitting BP was measured two times first with a 30s interval. If two measurements differed by <5 mmHg, BP was recorded as the mean of the two measurements. If two measurements differed by >5 mmHg, BP was remeasured and the final BP in each examination was calculated as the mean of three measurements. Hypertension in each examination was defined as SBP≥140 mmHg and/or DBP≥90 mmHg or BP<140 mmHg and DBP<90 mmHg with regular antihypertensive drugs usage. Individuals were regarded as having hypertension if they were recorded as having hypertension in at least two examinations.

### 2.4. BPV Calculation

BPV was calculated by three methods. (1) Standard deviation (SD) of the BP levels is obtained from the physical examinations. The SD of SBP was recorded as SSD, and the SD of DBP was recorded as DSD. (2) The coefficient of the variation of BP (CV) was calculated as SD/mean of BP levels obtained from the physical examinations *∗*100%. The CV of SBP was recorded as coefficient of the variation of SBP (SCV), and the CV of DBP was recorded as coefficient of the variation of DBP (DCV). (3) The variation independent of mean (VIM) was calculated as SD/(mean of BP levels obtained from the physical examinations)^x^ (x was derived from curve fitting).

BPV levels in the current study were calculated based on the BP levels measured every two years. Several previous studies have demonstrated that the two-year BP measurement interval can sufficiently reflect the BP fluctuation over time [32–37] and may have greater prognostic value than short-term variability (e.g., minute-to-minute and and hour-to-hour) or average BP [20, 21].

### 2.5. PTA Measurement

Trained professional staff performed audiometric testing in a sound-isolating room using the Otometrics MADSEN Xeta audiometer (GN Group Co., Ltd., Ballerup, Denmark). Air-conduction hearing thresholds were measured for each ear using pure tone at six frequencies (0.5, 1, 2, 3, 4, and 6 kHz). 1KHz was used as the first pure-tone frequency from an intensity of -20 dB. If no response was observed, 5dB was added each time until response is observed. Then pure-tone frequencies at 0.5, 2, 3, 4, and 6 KHz were measured. PTAs were measured at low, intermediate, and high frequencies, respectively. PTA of low frequency was calculated by the mean of PTA at 0.5 and 1 KHz. PTA of intermediate frequency was calculated by the mean of PTA at 1 and 2 KHz. PTA of high frequency was calculated by the mean of PTA at 3, 4, and 6 KHz [38]. The final PTA was recorded as the larger value among PTAs of the left and right ears. Hearing loss levels were defined as normal hearing (PTA ≤ 25 dB) and hearing loss (PTA >25 dB) [39].

The testing should begin at relatively low frequencies ranging from 0.5 to 1 KHz, because this frequency is easily heard by most patients and has the greatest test-retest reliability. After that, the hearing test is performed at frequencies ranging from 1 to 2 KHz, which represent the intermediate frequencies of speech range. Then, the hearing test is performed at high frequencies ranging from 3 to 6 KHz. In clinical settings, many factors, such as sound injury, ototoxic drugs, and senile auditory system degeneration, may firstly affect the function of basal gyrus of cochlea. Consequently, early manifestation is the change of high-frequency hearing threshold [40].

The design of the current study is cross-sectional rather than a cohort one and the aim of this study is to reflect the distribution of BPV at the specific time-point (the year of 2014) and uncover the correlation between BPV and hearing ability. Therefore, we only focus on the relationship between BPV and hearing ability instead of their causal relationship. Consequently, only one hearing measurement is sufficient to identify the correlation between BPV and hearing ability.

### 2.6. Statistical Methods

Data were entered in the terminal of each hospital and then uploaded to the computer room of the Kailuan General Hospital for storage in an Oracle 10.2g database. SPSS 13.0 statistical software was utilized for statistical analysis. Normally distributed measurement data were recorded as mean±SD. Trend test was used to compare differences of multiple groups. If the variance is homogeneous, the LSD test is used. If the variance is not homogeneous, Dunnett's T3 test is used. Categorical variables were described as percentages and compared by the chi-square test. Multivariate linear regression analysis was used to investigate the impacts of BPV on PTAs and hearing loss. The collinearity was analyzed using variance inflation factor (VIF). Multivariate logistic regression analysis was used to analyze the effect of each SD increase in different BPV measurements on hearing loss. Sensitivity analyses were performed by removing individuals with occupational noise exposure and individuals with hypertension, respectively. P<0.05 (bilateral) was considered as statistically significant.

## 3. Results

Among the 101510 workers who participated in the 2006-2007 health examination, a total number of 8875 subjects participated in at least three physical examinations and had complete pure-tone threshold measurement data. In the 8875 individuals, 229 were excluded for female gender (n=138), the history of head injury (n=36), the history of stroke (n=24), myocardial infarction, or history of atrial fibrillation (n=31). As a result, a total of 8646 participants were included in the final statistical analysis.

**Figure 1:**
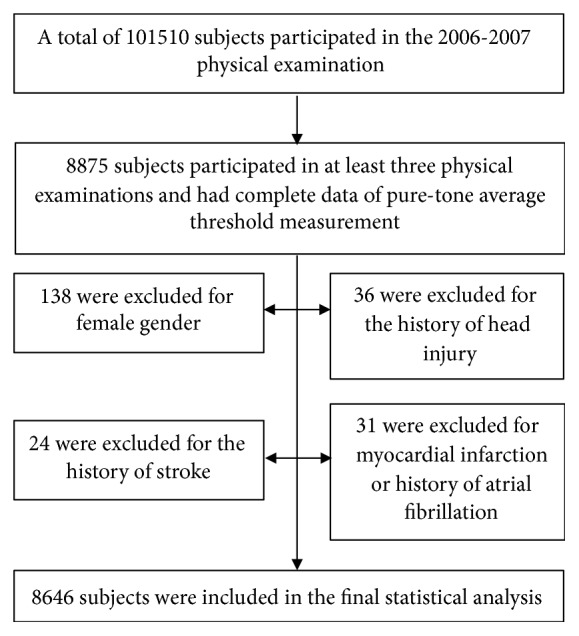
A flow chart of the current study.

As the Kailuan Group Corporation is a highly industrialized enterprise, the vast majority of the employees of the Kailuan Group Corporation are men (more than 80%). Moreover, the measurement of PTAs was performed on employees who work in coal mines. Since female employees rarely work in mines, the number of women is significantly less than that of men. Consequently, we excluded the 138 female subjects from further analyses. See Figure 1 for detailed information of participants' inclusions and exclusions.

### 3.1. Clinical Characteristics of Participants in Different BPV Groups

The 8646 participants were divided into four groups according to the quartiles of their SSD levels: (1) quartile 1 (n=2165): SSD<6.38; (2) quartile 2 (n=2156): 6.38≤SSD<9.07; (3) quartile 3 (n=2166): 9.07≤SSD<12.31; and (4) quartile 4 (n=2159): SSD≥12.31.  Table 1 summarized the clinical characteristics of participants in different BPV groups. With the increasing BPV levels (from quartile 1 to quartile 4), individuals were generally more likely to be older and presented with higher SBP, DBP, SSD, DSD, SCV, DCV, VIM_SBP_, VIM_DBP_, TC, higher hypertension and antihypertensive drug usage percentages, higher diabetes mellitus percentage, higher dyslipidemia percentage, and lower education percentage (p for trend <0.001).

### 3.2. Pure-Tone Average Thresholds (PTA) and Hearing Loss Distribution in Different BPV Groups

Participants' PTA values and hearing loss percentages in different BPV groups are shown in Table 2. PTAs of low, intermediate, and high frequency grew gradually with increasing BPV levels. Furthermore, significantly higher PTAs of low, intermediate, and high frequency were observed in individuals with highest BPV level (quartile 4) compared with individuals with lowest BPV level (quartile 1) (p for trend <0.001). Similarly, with the increasing BPV levels, the percentages of hearing loss in all three frequencies were generally increased (p for trend = 0.002 for low frequency; p for trend < 0.001 for intermediate and high frequency). As the BPV levels increased from quartile 1 to quartile 4, the PTA values and the percentages of hearing loss increased by 1.06 dB and 4.00%, 1.24 dB and 6.20%, and 2.37 dB and 7.10% at low, intermediate, and high frequency, respectively.

### 3.3. Multivariate Linear Regression Analysis between BPV and PTA

To identify the factors associated with PTA values, we performed a multivariate linear regression analysis with PTA as dependent variable. Independent variables included SSD, DSD, SCV, DCV, VIM_SBP_, and VIM_DBP_. In the analysis, we adjusted for BP level, age, BMI, FBG, TC, occupational noise exposure, cigarette smoking, alcohol consumption, physical exercise, and antihypertensive drug usage. Multivariate linear regression analysis between BPV and PTA is shown in Table 3.

The results showed that variations of SBP (SSD, SCV, and VIM_SBP_) were generally positively correlated with PTAs at low (P=0.035 for SCV), intermediate (p=0.024 for SSD; p=0.017 for SCV), and high frequency (p=0.003 for SSD; p=0.009 for SCV; p=0.029 for VIM_SBP_). However, for variations of DBP, no significant relationship was revealed between DSD, DCV, VIM_DBP_, and PTAs.

Because hypertension and antihypertensive drugs affect BPV levels, we further divided the individuals into nonhypertension group (n=5111) and hypertension group (n=3491) and reanalyzed the relationship between BPV and PTA by multivariate linear regression model. In nonhypertension group, the results showed that variations of SBP (SSD and SCV) were positively correlated with PTAs at intermediate (p=0.017 for SSD) and high frequency (p=0.004 for SSD; p=0.017 for SCV). However, no significant relationship was revealed in hypertension group. See Supplementary Table 1 for detailed information.

### 3.4. Multivariate Logistic Regression Analysis between BPV and Hearing Loss

To further demonstrate the relationship between BPV and hearing loss, multivariate logistic regression analysis was performed with the existence of hearing loss as dependent variable (0 = without hearing loss; 1 = with hearing loss). Independent variables included each SD increase of SSD, DSD, SCV, DCV, VIM_SBP_, and VIM_DBP_. In the multivariate logistic regression analysis, we adjusted for BP level, age, BMI, cigarette smoking, alcohol consumption, physical exercise, diabetes mellitus, dyslipidemia, antihypertensive drug usage, and occupational noise exposure. Multivariate logistic regression analysis between BPV and hearing loss is shown in Table 4.

The results indicated that each SD increase in SSD was positively associated with hearing loss at intermediate and high frequencies (OR (95% CI)=1.09 (1.02-1.17) for intermediate frequency; OR (95% CI)=1.07 (1.01-1.14) for high frequency). For CV, the results showed that each SD increase in SCV was positively associated with hearing loss at intermediate and high frequencies (OR (95% CI)=1.08 (1.01-1.15) for intermediate frequency; OR (95% CI)=1.06 (1.003-1.12) for high frequency). In terms of VIM, the results suggested that each SD increase in VIM_SBP_ was also positively associated with hearing loss at intermediate frequencies (OR (95% CI)=1.07 (1.004-1.14)). However, no correlation was found between DBP variations and hearing loss.

We also divided the individuals into nonhypertension group (n=5111) and hypertension group (n=3491) and reanalyzed the relationship between BPV and hearing loss by multivariate logistic regression model. In nonhypertension group, the results showed that variations of SBP (SSD and SCV) were positively correlated with hearing loss at intermediate (OR (95% CI)=1.12 (1.02-1.24) for SSD; OR (95% CI)=1.09 (1.00-1.19) for SCV) and high frequency (OR (95% CI)=1.11 (1.01-1.21) for SSD; OR (95% CI)=1.08 (1.001-1.17) for SCV). However, no significant relationship was observed in hypertension group. See Supplementary Table 2 for detailed information.

### 3.5. Sensitivity Analysis

As occupational noise exposure is one of the predominant risk factors for hearing loss [41]; in the sensitivity analysis, we removed 2077 individuals with history of noise exposure to rule out the impacts of noise on hearing loss. Sensitivity analysis is shown in Supplementary Table 7. After excluding individuals with noise exposure, variations of SBP (SSD, SCV, and VIM_SBP_) remain positively correlated with hearing loss at intermediate frequency. The relationship between SSD and hearing loss at low frequency gained significance, while the correlation between VIM_SBP_ and hearing loss at high frequency lost significance. For variations of DBP, no significant correlation was identified.

## 4. Discussion

In this large-scale population-based cross-sectional study, we firstly investigated the relationship between BPV and hearing and found that PTAs and percentages of hearing loss at low, intermediate, and high frequencies grew gradually with increasing BPV levels. After adjusting for multiple covariates, multivariate linear regression analysis demonstrated that variations of SBP (SSD, SCV, and VIM_SBP_) were all positively correlated with PTAs at intermediate and high frequencies. Logistic regression analysis identified that each SD increase in SSD, SCV, and VIM_SBP_ was also positively associated with hearing loss at intermediate and high frequencies. These results indicated that BPV indeed had a positive relationship with PTA and hearing loss, especially at intermediate and high frequencies.

Hypertension has long been regarded as one of the essential risk factors underlying pathophysiological processes of the cochlea from early in the twentieth century [42]. Several studies have reported on the influence of hypertension on hearing loss from both clinical and experimental aspects. A previous study investigated pure-tone audiometry results and BP levels in middle-aged subjects. The results showed that, in subjects with hearing loss, 46.8% had hypertension. However, in subjects with normal hearing levels, only 29.9% had hypertension. Nonconditional logistic regression indicated that arterial hypertension is an independent risk factor for hearing loss [22]. In another study, researchers compared the PTA levels in 150 hypertensive patients and 124 normotensive subjects. The results suggested that individuals with BP higher than 180/110 mmHg had higher PTA levels at high frequencies [23]. Several mechanisms underlying the association between BP and hearing levels have also been reported, such as the disturbance of the inner ear potassium recycling process due to the detrimental action of natriuretic hormone [43] and the decrease in the cochlear oxygen partial pressure [38]. Current evidence linking hypertension to sensorineural high-frequency cochlear hearing loss in humans may be confounded by other concomitant diseases or risk factors such as age, coronary artery disease, diabetes, obesity, hyperlipidemia, smoking, and noise exposure. Therefore, further research in this field is clearly needed. Before analyzing the correlation between BPV and hearing ability, we analyzed the relationship between BP levels and hearing ability firstly to confirm the consistency between our study and previous reports. Overall, the results demonstrated that the BP levels were positively associated with PTAs and hearing loss, which is in accordance with the results of previous studies (see Supplementary Tables 3–6 for detailed information). Consequently, based on these preanalyses, the correlation identified between BPV and hearing ability in the current study may be reliable and reproducible.

As occupational noise exposure is an important risk factor of hearing loss, we remove subjects with noise exposure and reanalyzed the relationship between BPV and hearing loss. We found that the correlation between each SD increase in SSD, SCV, and VIM_SBP_ and hearing loss at intermediate frequency was still significant, while the significance of VIM_SBP_ at high frequency diminished and failed to achieve statistical significance. Therefore, our finding suggested that BPV may predominantly impact hearing loss at intermediate frequency. Many factors, especially noise injury, may firstly affect the function of basal gyrus of cochlea and manifested as changes of high-frequency hearing threshold. Therefore, when the subjects with noise exposure were removed from the sensitivity analyses, the statistical significance of BPV and high-frequency hearing loss may be diminished. Since the intermediate-frequency hearing ability is less likely to be influenced by noise exposure, the correlation between BPV and intermediate-frequency hearing ability remained significant. Cochlear apex, as the region responsible for low- and intermediate-frequency hearing, has been demonstrated to be sensitive to hemodynamic changes [24, 39, 40]. As an indicator of hemodynamic stability, higher BPV is more likely to lead to unstable blood supply of cochlea, which may result in the impaired intermediate hearing ability. This may also be the reason why only intermediate frequency is significant in the sensitivity analyses. Moreover, despite the fact that the relationship between each SD increase in SSD, SCV, and VIM_SBP_ and hearing loss at intermediate frequency remains significant, the OR values of these correlations decreased. These results suggested that noise may partly contribute to hearing loss resulting from BPV. We speculate that the influence of BPV on the inner ear causes the cochlea to be more vulnerable to noise. Thus the impacts of noise exposure, to some extent, compromised the effect of BPV on hearing loss.

It has been reported by previous studies that greater BPV is associated with higher risk of target organ damage [12] and cardiovascular events [41, 42]. Although the relationship between hypertension and hearing levels has been widely investigated, no previous study has reported the impacts of BPV on hearing. Whether the deleterious influence of hypertension on hearing loss can be partly mediated by BPV and whether the BPV could contribute to hearing loss independent of mean BP levels remain unknown. In our current study, after removing individuals with hypertension, each SD increase in SSD, SCV, and VIM_SBP_ was positively associated with hearing loss at intermediate and high frequencies. Our results demonstrated that BPV can independently correlated with hearing and hearing loss, and this effect remains significant without the contribution of hypertension.

Cochlea, as the main hearing organ, is supplied by the labyrinthine artery and they are terminal arteries without collateral vessels. The hair cells of cochlea are movable cells and play an important role in the process of acoustic amplification. Their function requires a lot of energy. Therefore, the cochlear hair cells are extremely sensitive to ischemia [43]. As an indicator of hemodynamic stability, higher BPV is more likely to lead to unstable blood supply of cochlea, which may result in the death of hair cells and reduced hearing sensitivity. Thus, lowering BPV may be a novel target for preventing hearing loss.

This study has some limitations. First, the participants of the current study are all male subjects and thus the relationship between BPV and hearing among female individuals remains unknown. Second, we did not validate the damage of BPV on cochlea by cellular or animal models. Third, this observation was performed on Chinese population. Whether the results can be generalized to individuals of other ancestries warrants further investigations.

## 5. Conclusions

This is the first and largest-scale population-based study to analyze the relationship between long-term BPV and hearing. After adjusting for multiple factors, we found that variations of SBP (SSD, SCV, and VIM_SBP_) positively correlated with PTA at intermediate and high frequencies. Each SD increase in SBP variants all contributed to greater risk of hearing loss at intermediate and high frequencies. Results of our study may explain the effect of BPV on hearing.

## Figures and Tables

**Table 1:** Clinical characteristics of participants in different BPV groups.

	Quartile 1 (SSD<6.4)	Quartile 2 (6.4≤SSD<9.1)	Quartile 3 (9.1≤SSD<12.3)	Quartile 4 (SSD≥12.3)	p for trend
	(n=2165)	(n=2156)	(n=2166)	(n=2159)	
Age, year	45.2±8.6	46.6±8.1^a^	47.0±8.1^a^	47.9±8.3^abc^	<0.001

SBP (mmHg)	126±11	129±13^a^	131±15^ab^	137±20^abc^	<0.001

DBP (mmHg)	81±9	82±9^a^	83±10^ab^	86±12^abc^	<0.001

Mean of times (BP)	4.3±0.8	4.5±0.7^a^	4.5±0.7^a^	4.4±0.8^abc^	<0.001

SSD (mmHg)	5±1	8±1^a^	11±1^ab^	16±5^abc^	<0.001

DSD (mmHg)	5±3	6±3^a^	7±3^ab^	9±4^abc^	<0.001

SCV	3.8±1.0	6.3±0.6^a^	8.5±0.7^ab^	12.6±2.9^abc^	<0.001

DCV	6.6±3.1	7.2±3.0^a^	8.2±3.4^ab^	10.7±4.4^abc^	<0.001

VIM_SBP_	4.9±1.5	8.0±1.5^a^	10.8±1.3^ab^	15.7±3.6^abc^	<0.001

VIM_DBP_	5.5±2.7	6.0±2.6^a^	6.9±2.9^ab^	8.9±3.6^abc^	<0.001

BMI (kg/m^2^)	25.2±3.2	25.3±3.2	25.2±3.3	25.0±3.3	0.034

FBG (mmol/L)	5.5±1.5	5.6±1.5	5.6±1.6^a^	5.7±1.6^abc^	<0.001

TC (mmol/L)	4.8±1.4	5.0±1.3^a^	5.0±1.4^a^	5.0±1.4^a^	<0.001

Occupational noise exposure, n (%)	560 (25.9)	502 (23.6)	522 (24.3)	493 (23.1)	0.066

Cigarette smoking, n (%)	1094 (60.2)	1157 (62.9)	1138 (61.3)	1144 (62.6)	0.272

Alcohol consumption, n (%)	217 (10.7)	240 (11.7)	221 (10.7)	270 (13.2)	0.037

Physical exercise, n (%)	170 (8.4)	160 (7.8)	164 (8.0)	129 (6.3)	0.022

Hypertension, n (%)	521 (23.9)	747 (34.9)	878 (40.6)	1345 (62.1)	<0.001

Antihypertensive drug usage, n (%)	57 (2.6)	73 (3.4)	112 (5.2)	240 (11.1)	<0.001

Diabetes mellitus, n (%)	140 (6.6)	159 (7.5)	182 (8.6)	268 (12.6)	<0.001

Dyslipidemia, n (%)	1524 (70.0)	1549 (72.3)	1588 (73.6)	1651 (76.2)	<0.001

SBP, systolic blood pressure; DBP, diastolic blood pressure; mean of times (BP), mean times of blood pressure measurement; SSD, standard deviation of systolic blood pressure; DSD, standard deviation of diastolic blood pressure; SCV, coefficient of the variation of systolic blood pressure; DCV, coefficient of the variation of diastolic blood pressure; VIM_SBP_, systolic blood pressure variation independent of mean; VIM_DBP_, diastolic blood pressure variation independent of mean; FBG, fasting blood glucose; BMI, body mass index; TC, total cholesterol; a, p<0.05 compared with quartile 1; b, p<0.05 compared with quartile 2; c, p<0.05 compared with quartile 3.

**Table 2:** Pure-tone average thresholds (PTAs) and hearing loss distribution in different BPV groups.

		Quartile 1 (SSD<6.4)	Quartile 2 (6.4≤SSD<9.1)	Quartile 3 (9.1≤SSD<12.3)	Quartile 4 (SSD≥12.3)	p for trend
		(n=2165)	(n=2156)	(n=2166)	(n=2159)	
Pure-tone average threshold (PTA, dB)	Low frequency	20.0±9.3	20.3±9.6	20.6±10.5	21.1±10.8^a^	<0.001
	Intermediate frequency	20.8±10.7	21.3±11.0	21.6±11.5	22.1±12.1^a^	<0.001
	High frequency	26.1±19.2	26.9±19.5	27.1±19.9	28.5±21.1^a^	<0.001

Hearing loss, n (%)	Low frequency	267 (12.3)	334 (15.6)	302 (14.0)	354 (16.3)	0.002
	Intermediate frequency	334 (15.2)	400 (18.3)	386 (17.6)	470 (21.4)	<0.001
	High frequency	532 (24.1)	592 (27.1)	582 (26.5)	685 (31.2)	<0.001

a, p<0.05 compared with quartile 1.

**Table 3:** Multivariate linear regression analysis between BPV and PTA.

BPV	PTA at low frequency		PTA at intermediate frequency		PTA at high frequency	
	B value (95% CI)	p value	B value (95% CI)	p value	B value (95% CI)	p value
SSD	0.05 (0.00-0.11)	0.050	0.07 (0.09-0.13)	0.024	0.16 (0.05-0.27)	0.003

DSD	0.05 (-0.03-0.12)	0.195	0.05 (-0.03-0.14)	0.195	0.07 (-0.08-0.21)	0.356

SCV	0.08 (0.01-0.15)	0.035	0.10 (0.02-0.18)	0.017	0.19 (0.05-0.33)	0.009

DCV	0.03 (-0.03-0.10)	0.295	0.04 (-0.03-0.11)	0.298	0.04 (-0.08-0.17)	0.518

VIM_SBP_	0.04 (-0.02-0.09)	0.169	0.05 (-0.01-0.11)	0.115	0.12 (0.01-0.23)	0.029

VIM_DBP_	0.11 (-0.10-0.32)	0.319	0.08 (-0.16-0.32)	0.528	0.10 (-0.32-0.52)	0.642

BPV, blood pressure variation; PTA, pure-tone average threshold; SSD, standard deviation of systolic blood pressure; DSD, standard deviation of diastolic blood pressure; SCV, coefficient of the variation of systolic blood pressure; DCV, coefficient of the variation of diastolic blood pressure; VIM_SBP_, systolic blood pressure variation independent of mean; VIM_DBP_, diastolic blood pressure variation independent of mean. Multivariate linear regression analysis was performed with PTA as dependent variable. Independent variables included SSD, DSD, SCV, DCV, VIM_SBP_, and VIM_DBP_ as independent variables. In the multivariate linear regression analysis, we adjusted for BP level, age, BMI, FBG, TC, occupational noise exposure, cigarette smoking, alcohol consumption, physical exercise, and antihypertensive drug usage.

**Table 4:** Multivariate logistic regression analysis between BPV and hearing loss.

	BPV groups	PTA at low frequency	PTA at intermediate frequency	PTA at high frequency
		OR value (95% CI)	OR value (95% CI)	OR value (95% CI)
SD	SSD (+SD)	1.07 (0.99-1.15)	1.09 (1.02-1.17)	1.07 (1.01-1.14)
	DSD (+SD)	1.04 (0.97-1.12)	1.04 (0.98-1.11)	1.02 (0.96-1.08)

CV	SCV (+SD)	1.06 (0.99-1.14)	1.08 (1.01-1.15)	1.06 (1.003-1.12)
	DCV (+SD)	1.03 (0.96-1.11)	1.03 (0.97-1.10)	1.01 (0.95-1.07)

VIM	VIM_SBP_ (+SD)	1.05 (0.98-1.12)	1. 07(1.004-1.14)	1.05 (0.997-1.11)
	VIM_DBP_ (+SD)	1.02 (0.96-1.10)	1.03 (0.96-1.09)	1.00 (0.95-1.06)

BPV, blood pressure variation; PTA, pure-tone average threshold; SD, standard deviation; CV, coefficient of the variation; VIM, variation independent of mean. Multivariate logistic regression analysis was performed with the existence of hearing loss as dependent variable (0 = without hearing loss; 1 = with hearing loss). Independent variables included each SD increase of SSD, DSD, SCV, DCV, VIM_SBP_, and VIM_DBP_. In the multivariate logistic regression analysis, we adjusted for BP level, age, BMI, cigarette smoking, alcohol consumption, physical exercise, diabetes mellitus, dyslipidemia, antihypertensive drug usage, and occupational noise exposure.

## Data Availability

The population-based data used to support the findings of this study may be released upon application to the Kailuan General Hospital that can be contacted at drwusl@163.com.

## Supplementary Materials

Supplementary Materials Supplementary materials description: this file included a total number of six supplementary tables. In Supplementary Tables 1-2, we divided the individuals in the current study into nonhypertension group (n=5111) and hypertension group (n=3491) and the multivariate linear regression analysis between BPV and PTA and the multivariate logistic regression analysis between BPV and hearing loss were reanalyzed in Supplementary Table 1 and Supplementary Table 2, respectively. In Supplementary Tables 3-6, to confirm the relationship between BP level and hearing ability identified by previous studies, we analyzed the relationship between BP level and hearing ability based on the study population of our current study. Supplementary Table 3 described the clinical characteristics of participants in different BP groups. Supplementary Table 4 described pure-tone average thresholds (PTAs) and hearing loss distribution in different BP groups. Supplementary Table 5 represented multivariate linear regression analysis between BP and PTA. Supplementary Table 6 represented multivariate logistic regression analysis between BP and hearing loss.
