# Supplementary material for: Blood Pressure Variability Is Associated with Hearing and Hearing Loss: A Population-Based Study in Males
Source: Int J Hypertens. 2019 Feb 3;2019:9891025. doi: 10.1155/2019/9891025 (PMC6377956; doi:10.1155/2019/9891025)
Supplement: Supplementary Materials — description: this file included a total number of six supplementary tables. In Supplementary Tables 1-2, we divided the individuals in the current study into nonhypertension group (n=5111) and hypertension group (n=3491) and the multivariate linear regression analysis between BPV and PTA and the multivariate logistic regression analysis between BPV and hearing loss were reanalyzed in Supplementary Table 1 and Supplementary Table 2, respectively. In Supplementary Tables 3-6, to confirm the relationship between BP level and hearing ability identified by previous studies, we analyzed the relationship between BP level and hearing ability based on the study population of our current study. Supplementary Table 3 described the clinical characteristics of participants in different BP groups. Supplementary Table 4 described pure-tone average thresholds (PTAs) and hearing loss distribution in different BP groups. Supplementary Table 5 represented multivariate linear regression analysis between BP and PTA. Supplementary Table 6 represented multivariate logistic regression analysis between BP and hearing loss. [file 9891025.f1.docx]

**Supplementary Table 1. Multivariate linear regression analysis between BPV and PTA among non-hypertension and hypertension individuals.**

| BPV | PTA at low frequency | | PTA at intermediate frequency | | PTA at high frequency | |
| --- | --- | --- | --- | --- | --- | --- |
|  | B value (95%CI) | p value | B value (95%CI) | p value | B value (95%CI) | p value |
| Non-Hypertension (n=5155) | | | | | | |
| SSD | 0.08 (0.01-0.15) | 0.034 | 0.10 (0.02-0.18) | 0.017 | 0.21 (0.07-0.36) | 0.004 |
| DSD | 0.04 (-0.06-0.14) | 0.457 | 0.06 (-0.06-0.17) | 0.311 | 0.08 (-0.13-0.28) | 0.078 |
| SCV | 0.07(-0.02-0.16) | 0.116 | 0.09 (-0.01-0.19) | 0.069 | 0.21 (0.04-0.39) | 0.017 |
| DCV | 0.02 (-0.06-0.10) | 0.662 | 0.03 (-0.06-0.12) | 0.486 | 0.05 (-0.11-0.20) | 0.580 |
| VIM_SBP_ | 0.03 (-0.03-0.10) | 0.287 | 0.05 (-0.03-0.12) | 0.201 | 0.13 (-0.001-0.25) | 0.051 |
| VIM_DBP_ | 0.01(-0.08-0.10) | 0.315 | 0.03(-0.08-0.13) | 0.327 | 0.04(-0.14-0.22) | 0.678 |
| Hypertension (n=3491) | | | | | | |
| SSD | 0.02(-0.06-0.10) | 0.636 | 0.03(-0.07-0.12) | 0.558 | 0.08(-0.08-0.25) | 0.313 |
| DSD | 0.01(-0.11-0.12) | 0.908 | -0.02(-0.15-0.10) | 0.709 | -0.10(-0.33-0.12) | 0.359 |
| SCV | 0.04(-0.07-0.16) | 0.452 | 0.06(-0.07-0.19) | 0.384 | 0.14(-0.09-0.38) | 0.224 |
| DCV | 0.02(-0.09-0.12) | 0.765 | -0.01(-0.13-0.11) | 0.863 | -0.08(-0.29-0.12) | 0.431 |
| VIM_SBP_ | 0.05(-0.05-0.15) | 0.313 | 0.06(-0.05-0.18) | 0.261 | 0.14(-0.06-0.34) | 0.167 |
| VIM_DBP_ | 0.03(-0.10-0.16) | 0.151 | -0.002(-0.15-0.15) | 0.976 | -0.09(-0.36-0.17) | 0.494 |

BPV, blood pressure variation; PTA, pure-tone average threshold; SSD, standard deviation of systolic blood pressure; DSD, standard deviation of diastolic blood pressure; SCV, coefficient of the variation of systolic blood pressure; DCV, coefficient of the variation of diastolic blood pressure; VIM_SBP_, systolic blood pressure variation independent of mean; VIM_DBP_, diastolic blood pressure variation independent of mean; Multivariate linear regression analysis was performed with PTA as dependent variable. Independent variables included SSD, DSD, SCV, DCV, VIM_SBP_, and VIM_DBP_ as independent variables. In the multivariate linear regression analysis, we adjusted for BP level, age, BMI, FBG, TC, occupational noise exposure, cigarette smoking, alcohol consumption, physical exercise, and antihypertensive drug usage.

**Supplementary Table 2. Multivariate logistic regression analysis between BPV and hearing loss among non-hypertension and hypertension individuals.**

|  | BPV groups | PTA at low frequency | PTA at intermediate frequency | PTA at high frequency |
| --- | --- | --- | --- | --- |
|  |  | OR value (95%CI) | OR value (95%CI) | OR value (95%CI) |
| Non-Hypertension (n=5155) | | | | |
| SD | SSD (+SD) | 1.11 (0.995-1.24) | 1.12 (1.02-1.24) | 1.11 (1.01-1.21) |
|  | DSD (+SD) | 1.02 (0.91-1.14) | 1.03 (0.93-1.14) | 1.003 (0.92-1.09) |
| CV | SCV (+SD) | 1.08 (0.98-1.19) | 1.09 (1.00-1.19) | 1.08 (1.001-1.17) |
|  | DCV (+SD) | 1.01 (0.91-1.11) | 1.02 (0.93-1.12) | 0.99 (0.92-1.07) |
| VIM | VIM_SBP_ (+SD) | 1.06 (0.97-1.15) | 1. 07 (0.98-1.16) | 1.06 (0.99-1.14) |
|  | VIM_DBP_ (+SD) | 1.00 (0.91-1.10) | 1.01 (0.93-1.10) | 0.98 (0.91-1.06) |
| Hypertension (n=3491) | | | | |
| SD | SSD (+SD) | 1.02 (0.92-1.13) | 1.06 (0.97-1.16) | 1.03 (0.95-1.12) |
|  | DSD (+SD) | 1.02 (0.93-1.13) | 1.00 (0.92-1.09) | 0.99 (0.92-1.07) |
| CV | SCV (+SD) | 1.03 (0.93-1.15) | 1.07 (0.98-1.18) | 1.04 (0.96-1.14) |
|  | DCV (+SD) | 1.03 (0.93-1.14) | 1.01 (0.92-1.11) | 1.00 (0.92-1.08) |
| VIM | VIM_SBP_ (+SD) | 1.05 (0.94-1.17) | 1. 09 (0.99-1.21) | 1.05 (0.96-1.15) |
|  | VIM_DBP_ (+SD) | 1.04 (0.93-1.15) | 1.02 (0.92-1.12) | 1.00 (0.92-1.09) |

BPV, blood pressure variation; PTA, pure-tone average threshold; SD, standard deviation; CV, coefficient of the variation; VIM, variation independent of mean; Multivariate logistic regression analysis was performed with the exist of hearing loss as dependent variable (0=without hearing loss; 1=with hearing loss). Independent variables included each SD increase of SSD, DSD, SCV, DCV, VIM_SBP_, and VIM_DBP_. In the multivariate logistic regression analysis, we adjusted for BP level, age, BMI, cigarette smoking, alcohol consumption, physical exercise, diabetes mellitus, dyslipidemia, antihypertensive drug usage, and occupational noise exposure.

**Supplementary Table 3. Clinical characteristics of participants in different BP groups.**

|  | Normal | Elevated | Hypertension | p for  trend |
| --- | --- | --- | --- | --- |
|  | (n=3004) | (n=8160) | (n=3744) |  |
| Age, year | 38.8±9.3 | 41.6±9.3^a^ | 46.3±8.2^ab^ | <0.001 |
| SBP (mmHg) | 111.5±6.2 | 127.9±6.6^a^ | 147.3±12.3^ab^ | <0.001 |
| DBP (mmHg) | 70.0±5.7 | 79.9±6.0^a^ | 92.9±8.1^ab^ | <0.001 |
| BMI (kg/m^2^) | 23.5±3.0 | 24.9±3.2^a^ | 25.8±3.38^ab^ | <0.001 |
| FBG (mmol/L) | 5..1±0.9 | 5.4±1.3^a^ | 5.8±1.6^ab^ | <0.001 |
| TC (mmol/L) | 4.7±0.9 | 5.0±1.0^a^ | 5.3±1.1^ab^ | <0.001 |
| Occupational noise exposure, n (%) | 884 (29.4) | 2236 (27.4) | 947 (25.3) | <0.001 |
| Cigarette smoking, n (%) | 1740 (58.2) | 4780 (58.9) | 2198 (59.0) | 0.536 |
| Alcohol consumption, n (%) | 1087 (36.4) | 2780 (34.3) | 1455 (39.0) | 0.009 |
| Physical exercise, n (%) | 921 (34.9) | 2404 (31.9) | 1050 (29.8) | <0.001 |
| Antihypertensive drug usage, n (%) | 33 (1.1) | 206 (2.5) | 477 (12.7) | <0.001 |
| Diabetes mellitus, n (%) | 70 (2.4) | 428 (5.3) | 440 (11.8) | <0.001 |
| Dyslipidemia, n (%) | 1641 (54.6) | 5431 (66.6) | 2942 (78.6) | <0.001 |

SBP, systolic blood pressure; DBP, diastolic blood pressure; Mean of times (BP), mean times of blood pressure measurement; FBG, fasting blood glucose; BMI, body mass index; TC, total cholesterol; a, p<0.05 compared with the normal BP group; b, p<0.05 compared with the elevated BP group; c, p<0.05 compared with the hypertension group.

**Supplementary Table 4**. **Pure-tone average thresholds (PTAs) and hearing loss distribution in different BP groups.**

|  | | Normal | Elevated | Hypertension | p for trend |
| --- | --- | --- | --- | --- | --- |
|  |  | (n=3004) | (n=8160) | (n=3744) |  |
| Pure-tone average threshold  (PTA, dB) | Low frequency | 18.4±7.9 | 18.8±8.2 | 19.8±9.6 | <0.001 |
|  | Intermediate frequency | 18.8±9.0 | 19.4±9.2 | 20.7±10.8 | <0.001 |
|  | High frequency | 22.2±15.9 | 22.9±16.2 | 25.3±18.9 | <0.001 |
| Hearing loss, n (%) | Low frequency | 217 (7.2) | 689 (8.4) | 438 (11.7) | <0.001 |
|  | Intermediate frequency | 254 (15.1) | 868 (10.6) | 561 (15.0) | 0.001 |
|  | High frequency | 447 (14.9) | 1398 (17.1) | 829 (22.1) | <0.001 |

| model | BP | PTA at low frequency | | PTA at intermediate frequency | | PTA at high frequency | |
| --- | --- | --- | --- | --- | --- | --- | --- |
|  |  | B value (95%CI) | p value | B value (95%CI) | p value | B value (95%CI) | p value |
| Model 1 | SBP | 0.03 (0.02-0.14) | <0.001 | 0.04 (0.03-0.05) | <0.001 | 0.06 (0.04-0.08) | <0.001 |
|  | DBP | 0.05 (0.03-0.06) | <0.001 | 0.06 (0.04-0.07) | <0.001 | 0.10 (0.07-0.12) | <0.001 |
| Model 2 | SBP | 0.02 (0.01-0.03) | <0.001 | 0. 02 (0.01-0.03) | <0.001 | 0.03 (0.01-0.05) | 0.010 |
|  | DBP | 0.03 (0.02-0.04) | 0.001 | 0.03 (0.01-0.04) | 0.004 | 0.03 (0.01-0.07) | 0.026 |

**Supplementary Table 5. Multivariate linear regression analysis between BP and PTA.**

SBP, systolic blood pressure; DBP, diastolic blood pressure; Model 1, adjusted for age. Model 2, adjusted for age, BMI, FBG, TC, cigarette smoking, alcohol consumption, physical exercise, occupational noise exposure, and antihypertensive drug usage.

**Supplementary Table 6. Multivariate logistic regression analysis between BP and hearing loss.**

| BP groups | PTA at low frequency | PTA at intermediate frequency | PTA at high frequency |
| --- | --- | --- | --- |
|  | OR value (95%CI) | OR value (95%CI) | OR value (95%CI) |
| Normal | 1 | 1 | 1 |
| Elevated | 1.06 (0.89-1.25) | 1.15 (0.98-1.34) | 1.07 (0.94-1.21) |
| Hypertension | 1.21 (1.01-1.25) | 1.08 (1.01-1.15) | 1.17 (1.02-1.35) |
| SBP(+SD) | 1.09 (1.03-1.15) | 1.10 (1.05-1.16) | 1.07 (1.02-1.12) |
| DBP(+SD) | 1.11 (1.04-1.18) | 1.10 (1.04-1.16) | 1.09 (1.04-1.14) |

SBP, systolic blood pressure; DBP, diastolic blood pressure; SD, standard deviation. We adjusted for age, BMI, cigarette smoking, alcohol consumption, physical exercise, diabetes mellitus, dyslipidemia, antihypertensive drug usage, and occupational noise exposure.

**Supplementary Table 7. Sensitivity analysis.**

| N=6569 | BPV groups | PTA at low frequency | PTA at intermediate frequency | PTA at high frequency |
| --- | --- | --- | --- | --- |
|  |  | OR value (95%CI) | OR value (95%CI) | OR value (95%CI) |
| SD | SSD (+SD) | 1.09 (1.003-1.19) | 1.11 (1.03-1.20) | 1.08 (1.01-1.16) |
|  | DSD (+SD) | 1.07 (0.99-1.16) | 1.05 (0.98-1.13) | 1.04 (0.98-1.11) |
| CV | SCV (+SD) | 1.08 (0.99-1.17) | 1.09 (1.02-1.18) | 1.07 (1.00-1.14) |
|  | DCV (+SD) | 1.06 (0.98-1.15) | 1.04 (0.97-1.12) | 1.03 (0.97-1.10) |
| VIM | VIM_SBP_ (+SD) | 1.06 (0.98-1.15) | 1.08 (1.002-1.16) | 1.05 (0.99-1.12) |
|  | VIM_DBP_ (+SD) | 1.06 (0.97-1.14) | 1.04 (0.97-1.12) | 1.03 (0.96-1.09) |

Sensitivity analysis was performed by removing individuals with occupational noise exposure; BPV, blood pressure variation; PTA, pure-tone threshold average; SD, standard deviation; CV, coefficient of the variation; VIM, variation independent of mean; As occupational noise exposure is one of the predominant risk factors for hearing loss, in the sensitivity analysis, we removed individuals with history of noise exposure to rule out the impacts of noise on the occurrence of hearing loss. Multivariate logistic regression analysis was performed with the exist of hearing loss as dependent variable (0=without hearing loss; 1=with hearing loss). Independent variables included each SD increase of SSD, DSD, SCV, DCV, VIM_SBP_, and VIM_DBP_. In the multivariate logistic regression analysis, we adjusted for BP level, age, BMI, cigarette smoking, alcohol consumption, physical exercise, diabetes mellitus, dyslipidemia, and antihypertensive drug usage.
